# Two new species of *Tricholomopsis* (Agaricales) from China based on molecular phylogenetic and morphological evidence

**DOI:** 10.3897/mycokeys.131.185587

**Published:** 2026-04-24

**Authors:** Gengshen Wang, Ting Guo, Xuan Chen, Jinpeng Liao, Yunrui Ma, Cong Huang, Cuixin Li

**Affiliations:** 1 College of Biological Science and Food Engineering, Southwest Forestry University, Kunming 650224, China Kunming Institute of Botany, Chinese Academy of Sciences Kunming China; 2 Forest Resources Exploitation and Utilization Engineering Research Center for Grand Health of Yunnan Provincial Universities, Southwest Forestry University, Kunming 650224, China Forest Resources Exploitation and Utilization Engineering Research Center for Grand Health of Yunnan Provincial Universities, Southwest Forestry University Kunming China; 3 Yunnan Key Laboratory for Fungal Diversity and Green Development, Kunming Institute of Botany, Chinese Academy of Sciences, Kunming, China College of Biological Science and Food Engineering, Southwest Forestry University Kunming China; 4 Edible Fungi Research Institute, Shanghai Academy of Agricultural Sciences, Shanghai, China Shanghai Academy of Agricultural Sciences Shanghai China; 5 Ministry of Agriculture Key Laboratory for Southern Edible Fungi Resources Utilization, Shanghai, China National Engineering Technology Development Center for Edible Fungi Shanghai China; 6 National Engineering Technology Development Center for Edible Fungi, Shanghai, China Ministry of Agriculture Key Laboratory for Southern Edible Fungi Resources Utilization Shanghai China; 7 Management Bureau of Tianbaoyan National Nature Reserve in Yong’an, Fujian Province, China Management Bureau of Tianbaoyan National Nature Reserve in Yong’an Fujian China; 8 Yunnan Junshijie Biotechnology Ltd., Yunnan (Rare Edible Fungi) Enterprise Technology Center, Kunming, China Yunnan Junshijie Biotechnology Ltd., Yunnan (Rare Edible Fungi) Enterprise Technology Center Kunming China; 9 Yunnan Junshijie Biotechnology Ltd., Kunming (Edible Fungi) Enterprise Technology Center, Kunming, Yunnan, China Yunnan Junshijie Biotechnology Ltd.,Kunming (Edible Fungi) Enterprise Technology Center Kunming China; 10 Management Center of the Zhongjianhe National Nature Reserve for Chinese Giant Salamander, Xianfeng County, Hubei, China Management Center of the Zhongjianhe National Nature Reserve for Chinese Giant Salamander Xianfeng China

**Keywords:** Morphology, molecular phylogeny, Phyllotopsidaceae, taxonomy

## Abstract

*Tricholomopsis* is a genus of tricholomoid fungi, typically saprotrophic, characterized by a fibrillose to squamulose pileus, yellow lamellae, and prominent cheilocystidia. In this study, two new species of *Tricholomopsis*, namely *T. roseorubra* and *T. cortinata*, are described based on morphological characteristics and phylogenetic analyses of ITS–28S sequences. *Tricholomopsis roseorubra* is characterized by purplish-red, squamulose basidiomata and prominent cheilocystidia forming a sterile gill edge, whereas *T. cortinata* is distinguished by small basidiomata, globose basidiospores, and a well-developed cortinate annulus. The latter represents the first record of a cortinate annulus in *Tricholomopsis*.

## Introduction

*Tricholomopsis* (Singer 1939) is a morphologically distinctive genus of agaricoid fungi characterized by tricholomoid basidiomata with often brightly colored pilei, yellow lamellae, smooth and non-amyloid basidiospores, and well-developed cheilocystidia frequently forming a sterile lamellar edge. The pileipellis is typically arranged as a cutis to trichoderm. Species of *Tricholomopsis* are predominantly lignicolous and saprotrophic species that occur on decaying wood or forest litter in temperate to montane ecosystems, including bamboo-dominated habitats (Singer 1953; Smith 1960; Wang et al. 2024). To date, more than 40 species have been described worldwide, with records spanning Africa, Asia, Australia, Europe, North America, and South America (Singer 1939, 1943, 1953, 1989; Thiers 1958; Hongo 1959, 1960, 1966; Smith 1960; Horak 1971, 1980; Pegler 1977, 1983; Garrido 1988; He 1989; Singer et al. 1990; Liu 1994; Holec 2009; Olariaga et al. 2015; Cooper and Park 2016; Desjardin 2017; Holec et al. 2019; Mao et al. 2021; Wang and Yang 2023; Wang et al. 2024; Hyde et al. 2024). In China, approximately 16 species have been confirmed based on morphological and molecular evidence (Hosen et al. 2020; Mao et al. 2021; Liu et al. 2022; Wang and Yang 2023; Wang et al. 2024).

The systematic placement of *Tricholomopsis* has long been controversial. It was traditionally assigned to *Tricholomataceae* based primarily on macromorphological similarities (Singer 1939). However, recent multi-locus phylogenetic and phylogenomic studies have consistently demonstrated that *Tricholomopsis* belongs to *Phyllotopsidaceae*, together with *Phyllotopsis*, *Pleurocybella*, *Conoloma*, and *Neotricholomopsis* (Fan et al. 2024; Vizzini et al. 2024; Wang et al. 2024). Within this phylogenetic framework, several well-supported infrageneric lineages have been recognized and formalized as sections, including sect. *Tricholomopsis*, sect. *Decoramentum*, sect. *Glabrae*, and sect. *Bambusinae* (Wang et al. 2024).

During field surveys of macrofungi in bamboo-dominated forests of China, two previously undescribed species of *Tricholomopsis* were collected and are here described as new species based on detailed morphological observations and phylogenetic analyses of a concatenated ITS–28S dataset.

## Materials and methods

### Specimens and morphological study

Specimens studied in this research were collected from Yunnan Province, Fujian Province, and Shanghai, China. Voucher specimens were deposited in the Cryptogamic Herbarium of Kunming Institute of Botany, Chinese Academy of Sciences (**KUN-HKAS**), and the Herbarium of the Southwest Forestry University (**SWFC**).

Macroscopic descriptions were based on field notes and images, and terms were referred to Vellinga and Noordeloos (2001). Color codes were based on Kornerup and Wanscher (1981). Micro-morphology was observed with light microscopy under a ZEISS Axiostar Plus microscope from the dried specimens. Thin sections were mounted in 5% KOH solution, and amyloidity was tested with Melzer’s reagent. In the descriptions of basidiospores, the abbreviation [*n/m/p*] means *n* basidiospores measured from *m* basidiomata of *p* collections; dimensions for basidiospores are given using a range notation of the form (a–)b–c(–d). The range *b–c* contains a minimum of 90% of the measured values. Extreme values, *a* or *d*, are given in parentheses. *Q* represents the “length/width ratio” of a basidiospore in the side view. “*Q*_av._” is the average *Q* ± standard deviation. The generic names used in this study are abbreviated as follows: “*C.*” for *Conoloma*, “*Ph.*” for *Phyllotopsis*, “*Pl.*” for *Pleurocybella*, and “*T.*” for *Tricholomopsis*.

### DNA extraction, PCR amplification, and sequencing

Total genomic DNA was extracted from specimens dried in silica gel using an Ezup Column Fungi Genomic DNA Purification Kit (Sangon Biotech, Shanghai, China). Primer pairs ITS1F/ITS4 and LR0R/LR5 were employed to amplify and sequence the internal transcribed spacers 1 and 2 along with 5.8S rDNA (ITS) and the D1–D3 domains of nuclear 28S rDNA (28S), respectively. The PCR reactions were performed in a 25 μL volume containing 12.5 μL 1× Taq PCR MasterMix, 9.5 μL ddH_2_O, 1 μL forward primers, 1 μL reverse primers, and 1 μL template DNA. Amplification was conducted under the following conditions: initial denaturation at 94 °C for 5 min, followed by 35 cycles of 94 °C for 30 s, 52 °C (for both ITS and 28S) for 45 s, and 72 °C for 1 min, with a final extension at 72 °C for 10 min. PCR products were checked by electrophoresis on 1% agarose gels and sent to a commercial sequencing facility (Sangon Biotech, Shanghai, China) for bidirectional Sanger sequencing. Sequences were assembled and manually corrected using BioEdit v7.0.9.0, then compared with reference data in GenBank via BLAST to confirm taxonomic identity. All newly generated sequences were uploaded to GenBase (National Genomics Data Center, NGDC).

### Phylogenetic analyses

Sequences of ITS and 28S obtained in this study and retrieved from GenBank are listed in Table 1. *Pleurocybella porrigens* and *Phyllotopsis nidulans* were chosen as outgroups (Wang et al. 2024). Two gene matrices consisting of ITS and 28S, respectively, were automatically aligned using MAFFT v7 (Katoh and Standley 2013) under the L-INS-i method and then manually refined in BioEdit (Hall 1999) to resolve ambiguities. The two matrices were then concatenated with raxmlGUI v2.0.16 (Edler et al. 2021). File format conversion for subsequent analyses was performed using ALTER (Glez-Peña et al. 2010). MrModelTest v2.3 (Nylander 2004) was used to select the best-fit nucleotide substitution model for each gene partition under the BIC criterion. Maximum likelihood (ML) analysis was performed with raxmlGUI v2.0.16, and support values were estimated by performing 1,000 rapid bootstrap replicates. BI analysis was performed using the Markov chain Monte Carlo method in MrBayes v3.2 (Ronquist et al. 2012) with three independent runs under the GTR+I+G substitution model for both ITS and 28S partitions. Each run was performed with four chains for two million generations, with sampling every 100 generations. Chain convergence was determined using Tracer v1.7 (Rambaut et al. 2018) to ensure sufficiently large effective sample sizes (>200). The consensus trees and Bayesian posterior probability values were obtained using the sump and sumt commands, in which the first 25% of generations were discarded as burn-in.

**Table 1.** Sequences used in this study and their accession numbers.

Taxon	Voucher	Locality	ITS	28S	Reference
*Conoloma mucronatum*	HKAS 125778	China: Yunnan	OP627092	OP604166	Wang and Yang (2023)
*C. mucronatum*	HKAS 125751 (Holotype)	China: Yunnan	OP627093	OP604167	Wang and Yang (2023)
*Phyllotopsis nidulans*	HMJAU 7272	China	OQ674789	OQ674464	Wang et al. (2023)
*Pleurocybella porrigens*	UPS F-611822	–	MT232355	MT232309	Unpublished
*Tricholomopsis aculeata*	HKAS 129330	China: Yunnan	ON641574	ON627795	Wang et al. (2023)
*T. aculeata*	HKAS 129331	China: Yunnan	ON641575	ON627794	Wang et al. (2023)
*T. aff. rutilans*	TUB 011582	German	KP058981	DQ071830	Olariaga et al. (2015)
*T. aff. rutilans*	UPS F-646220	Sweden	KP058984	KP058985	Olariaga et al. (2015)
*T. alborufescens*	PC0125126 (Holotype)	France	MT117052	–	Larue (2020)
*T. aurea*	SFSU: BAP 618	Africa	MF100961	MF100994	Desjardin (2017)
*T. aurea*	SFSU: DED 8327	Africa	MF100960	MF100960	Desjardin (2017)
*T. badinensis*	PRM 946195 (Holotype)	Slovakia	LS992163	LS992163	Holec et al. (2019)
*T. badinensis*	HKAS54874	China: Yunnan	MN918502	MN912468	Wang et al. (2023)
*T. badinensis*	HKAS 82995	China: Xizang	MN918503	MN912469	Wang et al. (2023)
*T. bambusina*	HKAS 129325	China: Jiangxi	ON641579	ON627790	Wang et al. (2023)
*T. bambusina*	HKAS 129334	China: Zhejiang	ON641580	ON627789	Wang et al. (2023)
*T. campestris*	HKAS 98074	China: Yunnan	ON641570	ON627786	Wang et al. (2023)
*T. campestris*	HKAS 116178	China: Xizang	ON641571	ON627787	Wang et al. (2023)
***T. cortinata***	HKAS 151682 (holotype)	China: Fujian	AA136285	AA136290	This study
***T. cortinata***	HKAS 151683	China: Shanghai	AA136286	AA136291	This study
***T. cortinata***	HKAS 151684	China: Shanghai	AA136284	AA136292	This study
***T. cortinata***	HKAS 151685	China: Shanghai	AA136287	AA136293	This study
*T. darjeelingensis*	CUHAM 119	India	OQ913610	OQ861109	Thapa et al. (2023)
*T. decora*	TENN 59574	USA: Arkansas, Johnson	AY329597	–	Unpublished
*T. decora*	AFTOL-ID 537	USA	DQ404384	AY691888	Olariaga et al. (2015)
*T. depressa*	HKAS 87884	China: Xizang	ON641550	ON627738	Wang et al. (2023)
*T. depressa*	HKAS 46097	China: Xizang	ON641551	ON627737	Wang et al. (2023)
*T. depressa*	HKAS 50387	China: Yunnan	ON641552	ON627740	Wang et al. (2023)
*T. flammula*	PRM 899180	Slovakia: Dobročský prales	FN554893	FN554893	Holec and Kolařík (2011)
*T. flammula*	HKAS 73509	China: Hubei	ON641546	ON627746	Wang et al. (2023)
*T. flava*	HKAS 96940 (Holotype)	China: Yunnan	MN918505	MN912471	Wang et al. (2023)
*T. flava*	HKAS 90527	China: Yunnan	MN918506	MN912473	Wang et al. (2023)
*T. flavescens*	NYSf 1195.1 (Syntype)	USA	UDB022699	UDB022699	Saar and Voitk (2015)
*T. floccosa*	HMJAU 23667	–	ON641508	ON627733	Wang et al. (2023)
*T. floccosa*	HMJAU 7497	–	ON641509	ON627732	Wang et al. (2023)
*T. floccosa*	HKAS 57681 (Holotype)	China: Yunnan	ON641510	ON627731	Wang et al. (2023)
*T. galeata*	BJTC FM1107 (Holotype)	China: Shanxi	MW871732	MW871622	Mao et al. (2021)
*T. glabra*	HKAS 129333	China: Hunan	ON641520	ON627799	Wang et al. (2023)
*T. glabra*	HKAS 129332 (Holotype)	China: Shandong	ON641521	ON627800	Wang et al. (2023)
*T. lechatii*	PAM-2022a	French Guiana	OM793061	OM793062	Jayawardena et al. (2022)
*T. mitirubicunda*	BJTC FM649 (Holotype)	China: Shanxi	MW871620	MW871823	Mao et al. (2021)
*T. mitirubicunda*	HKAS 59486	China: Yunnan	ON641527	ON627759	Wang et al. (2023)
*T. ornaticeps*	PDD 102769	New Zealand	KY010824	KY010828	Cooper and Park (2016)
*T. ornaticeps*	PDD 102517	New Zealand	KY010822	–	Cooper and Park (2016)
*T. osiliensis*	PRM 899461 (Isotype)	Estonia: Saaremaa	HE649943	HE649943	Vauras 2009; Holec (2012)
*T. pallidolutea*	HAS 398	China: Shanxi	MW871614	MW871640	Mao et al. (2021)
*T. pallidolutea*	BJTC FM 1184 (Holotype)	China: Shanxi	MW871749	MW871631	Mao et al. (2021)
*T. pallidolutea*	HKAS 129339	China: Xinjiang	ON641572	ON627756	Wang et al. (2023)
*T. pteridiicola*	ARAN-Fungi 00321	Spain: Gipuzkoa, Artaleku	KP058992	KP058993	Olariaga et al. (2015)
*T. pteridiicola*	HKAS 129420	Austria	ON641526	ON627791	Wang et al. (2023)
***T. roseorubra***	HKAS 151680 (Holotype)	China: Yunnan	AA136282	AA136288	This study
***T. roseorubra***	HKAS 151681	China: Yunnan	AA136283	AA136289	This study
*T. rubroaurantiacus*	GDGM 61492 (Holotype)	China: Guangdong	MN912495	MN912492	Hosen et al. (2020)
*T. rubroaurantiacus*	HKAS 129324	China: Guangdong	ON641573	ON627792	Wang et al. (2023)
*T. rutilans*	HKAS 69507	China: Yunnan	ON641565	ON627754	Wang et al. (2023)
*T. rutilans*	HKAS 50428	China: Yunnan	ON641566	ON627752	Wang et al. (2023)
*T. rutilans* var. *splendidissima*	LIP86127 (Holotype)	France: La Chaise-Dieu	KP058995	–	Olariaga et al. (2015)
*T. sasae*	HKAS 87193	China: Yunnan	ON641576	–	Wang et al. (2023)
*T. sasae*	HKAS 31210	China: Hunan	ON641577	ON627793	Wang et al. (2023)
*T. scabra*	PDD 102579 (Holotype)	New Zealand: North Island	KY010823	KY010832	Cooper and Park (2016)
*T. scabra*	PDD 102100	New Zealand	KY010821	KY010831	Cooper and Park (2016)
*T.* sp.	MEL: 2382955	Australia: NT, Howard Springs	KP012816	–	Unpublished
*T.* sp.	MEL: 2382784	Australia: NT, Berry Springs	KP012965	KP012965	Unpublished
*T. sulphureoides*	NYSf 3116.1 (Holotype)	USA: New York State	UDB023122	UDB023122	Saar and Voitk (2015)
*T. yunnanensis*	HKAS 39166	China: Yunnan	ON641542	–	Wang et al. (2023)
*T. yunnanensis*	HKAS 76311	China: Sichuan	ON641536	ON627775	Wang et al. ( 2023)
*T. yunnanensis*	HKAS 56922	China: Yunnan	ON641537	ON627774	Wang et al. (2023)

## Results

### Phylogenetic analyses

**Figure 1.**
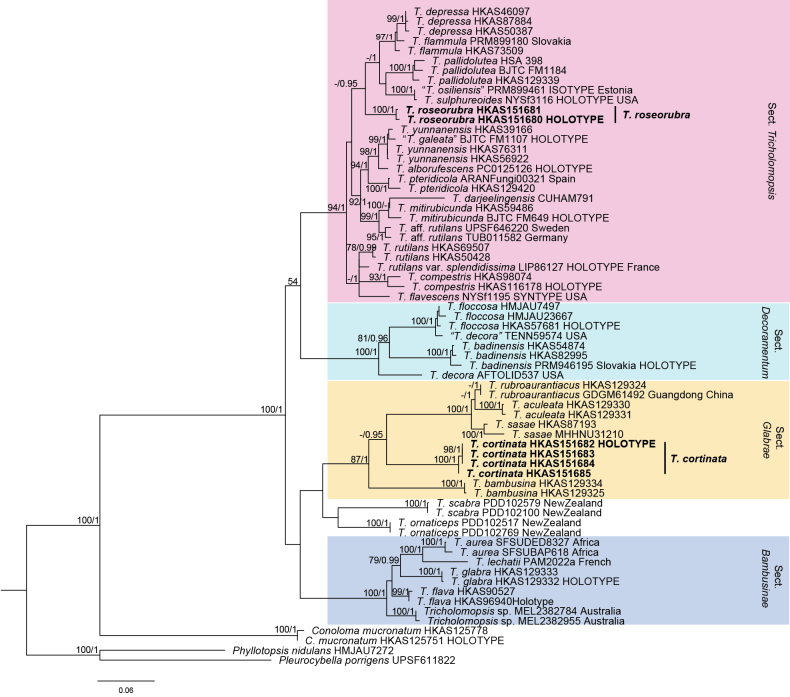
Maximum-likelihood phylogenetic tree of *Tricholomopsis* based on the concatenated ITS–28S dataset. Bootstrap values over 70% and Bayesian posterior probabilities over 0.90 are shown along branches. Two new species are highlighted in bold face.

In this study, six new sequences of ITS and six of 28S were generated. Finally, a total of 67 ITS and 54 28S sequences from 28 species of *Tricholomopsis* were analyzed (Table 1). The aligned matrix of ITS contains 760 bp, of which 331 are conserved sites, 409 are variable sites, and 333 are parsimony-informative sites. Whereas the aligned matrix of 28S contains 952 bp, of which 663 are conserved sites, 261 are variable sites, and 171 are parsimony-informative sites. The phylogenetic trees reconstructed from ML and BI analyses were of similar topologies, with high congruence in branching patterns and strong support at key nodes. Given that, only the tree inferred from ML analysis is shown (Fig. 1). Both analyses consistently recovered *Tricholomopsis* as a monophyletic genus with high bootstrap and posterior probability values. Most species formed well-supported clades corresponding to known taxonomic sections. However, samples collected in this study formed two independent lineages, of which one clustered within sect. *Tricholomopsis* and the other fell within sect. *Bambusina*.

### Key to the species of *Tricholomopsis* in China

**Table:** 

1	Basidiomata occurring on bamboo substrates; pleurocystidia absent or rare	**2**
–	Basidiomata not occurring on bamboo substrates; pleurocystidia present	**8**
2	Stipe with a well-developed cortinate annulus; basidiospores globose to subglobose	***T. cortinata***
–	Stipe lacking a cortinate annulus; basidiospores ellipsoid, ovoid, or subglobose	**3**
3	Pileus surface smooth, glabrous, or with inconspicuous scales	**4**
–	Pileus surface distinctly covered with scales	**5**
4	Pleurocystidia absent; cheilocystidia present	***T. flava***
–	Pleurocystidia present; cheilocystidia absent	***T. glabra***
5	Pileus dark yellow; scales dark red-brown	***T. bambusina***
–	Pileus light yellow to light orange; scales reddish-brown	**6**
6	Pileus light yellow; basidiospores ellipsoid to ovoid	***T. sasae***
–	Pileus light orange; basidiospores subglobose to broadly ellipsoid	**7**
7	Stipe > 4 cm long; basidiospores > 6 μm long	***T. aculeata***
–	Stipe < 4 cm long; basidiospores < 6 μm long	***T. rubroaurantiaca***
8	Pileus surface covered with grayish minute scales	**9**
–	Pileus surface covered with brownish, purplish, or reddish scales	**11**
9	Stipe > 8 mm wide; basidiospores subglobose to ellipsoid	***T. floccosa***
–	Stipe < 8 mm wide; basidiospores ellipsoid	**10**
10	Scales minute and pale; pileus dark yellow	***T. badinensis***
–	Scales coarse and dark; pileus bright yellow	***T. decora***
11	Pileus with brownish or dark scales; stipe < 5 mm wide	**12**
–	Pileus with purplish, grayish-rose to ruby-red, or red scales; stipe usually ≥ 5 mm wide	**13**
12	Basidiospores broadly ellipsoid to ellipsoid; pleurocystidia < 50 μm long	***T. sulphureoides***
–	Basidiospores ellipsoid to fusiform; pleurocystidia > 50 μm long	***T. pallidolutea***
13	Basidiomata small to medium-sized; cheilocystidia often containing brown vacuolar pigments	***T. roseorubra***
–	Basidiomata medium to large; cheilocystidia hyaline	**14**
14	Scales mealy, warped; stipe slender (< 1 cm wide)	**15**
–	Scales tomentose or adpressed; stipe robust (≥ 1 cm wide)	**16**
15	Pileus depressed; basidiospores wider than 4.5 μm	***T. depressa***
–	Pileus applanate; basidiospores narrower than 4.5 μm	***T. flammula***
16	Growing on buried litter in meadows; scales dark blond to orange	***T. campestris***
–	Growing on rotten wood or forest litter	**17**
17	Pleurocystidia absent or scarce	***T. rutilans***
–	Pleurocystidia present or abundant	**18**
18	Stipe densely covered with scales; scales purple-red	***T. yunnanensis***
–	Stipe sparsely covered with scales; scales brown	***T. mitirubicunda***

### Taxonomy

#### Tricholomopsis roseorubra

Taxon classificationFungiAgaricalesTricholomataceae

G.S. Wang sp. nov.

1EA175D1-E94F-5873-BD15-FEF2AB1EA734

Fungal Names: FN 573153

##### Etymology.

“roseo” = rose, “rubra” = red, referring to rose and ruby-red squamules on both pileus and stipe.

##### Diagnosis.

*Tricholomopsis roseorubra* is characterized by grayish-rose to ruby-red with fine squamules pileus and stipe, relatively small basidiomata, ellipsoid basidiospores, and cheilocystidia often containing brown vacuolar pigments. Solitary to gregarious on soil among decaying arrow-bamboo leaf litter at 3000–3200 m altitude.

**Figure 2.**
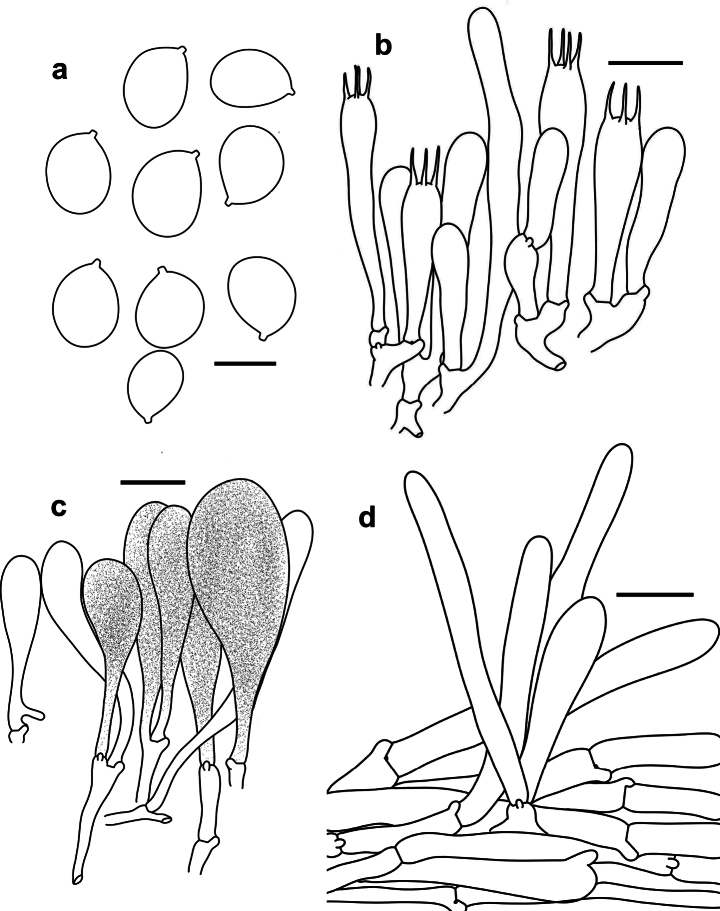
Microscopic features of *Tricholomopsis roseorubra* (HKAS 151680, holotype). **a**. Basidiospores; **b**. Hymenium and subhymenium; **c**. Cheilocystidia; **d**. Pileipellis. Scale bars: 5 μm (**a**), 10 μm (**b**), 20 μm (**c, d**).

##### Holotype.

China, • Yunnan Province: Nujiang Lisu Autonomous Prefecture, Lushui County, Pianma Township, altitude 3141 m, 24 Aug 2020, Yunrui Ma 300 (KUN-HKAS 151680); GenBank ITS: AA136282, 28S: AA136288.

##### Description.

Basidioma small to medium-sized. Pileus 2.3–7.5 cm, at first paraboloid to hemispherical, then plano-convex; surface dry, yellowish white to pale yellow (1A2–1A3), with grayish pink to grayish ruby (12B4–12B6, 12C4–12C6) scales. Lamellae sinuate to adnate, up to 4 mm broad, crowded, pale yellow to pastel yellow (1A2–1A4) with abundant lamellulae. Stipe 3–7 cm long, 0.2–0.7 cm thick, subcylindrical to cylindrical, pale yellow (1A2–1A3) with grayish pink to grayish ruby (12B4–12B6, 12C4–12C6) scales. Context of cap and stipe pale yellow (1A2–1A3).

Basidiospores [60/3/2] 6.5–7.2(–7.5) × 5.0–5.2(–5.5) μm, Q = 1.30–1.42(–1.44), Q_av._ = 1.38 ± 0.04, ellipsoid, thin-walled colorless and hyaline, smooth, non-amyloid; apiculus small. Basidia 20–30 × 5–7 μm, mostly 4-spored, occasionally 2-spored, narrowly clavate to clavate, thin-walled, sterigmata 4–5 μm. Cheilocystidia 40–75 × 10–32 μm, clavate, colorless hyaline or with brown vacuolar pigments, abundant, forming a sterile belt along the lamellar edge. Pleurocystidia 40–60 × 5.5–6.5 μm, cylindrical to flexuose, colorless hyaline, rare to absent. Pileipellis a cutis with the transition to a trichoderm at regular intervals, 30–50 μm thick and composed of radially arranged, thin-walled hyphae 5–12 μm wide, with brown pigments. Clamp connections present in all parts of basidioma.

**Figure 3.**
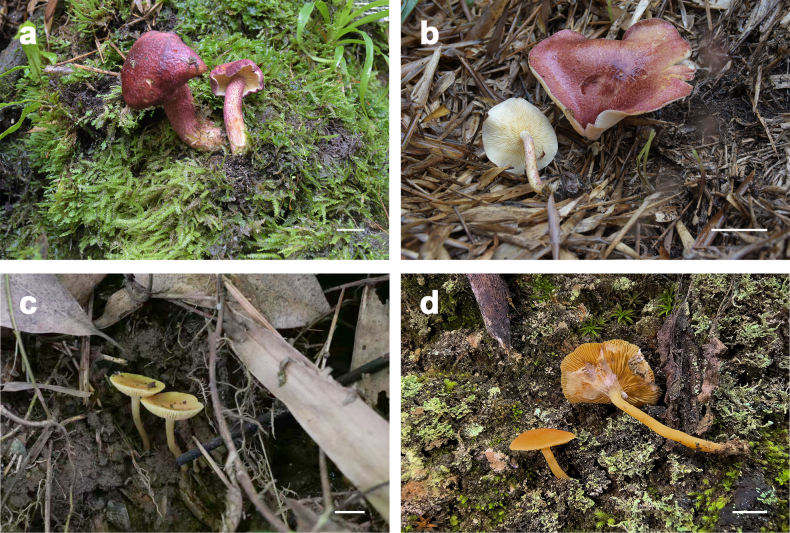
**a, b**. *Tricholomopsis roseorubra* (HKAS 151680 and HKAS 151681, photo by Yun-Rui Ma); **c, d**. *Tricholomopsis cortinata* (HKAS 151682, photo by Jin-Peng Liao; HKAS 151683, photo by Ting Guo). Scale bars: 1 cm (**a–d**).

##### Habitat and distribution.

Solitary to gregarious on soil among thick, decaying bamboo leaf litter in montane bamboo forest. Currently known from southwestern China (this study).

**Figure 4.**
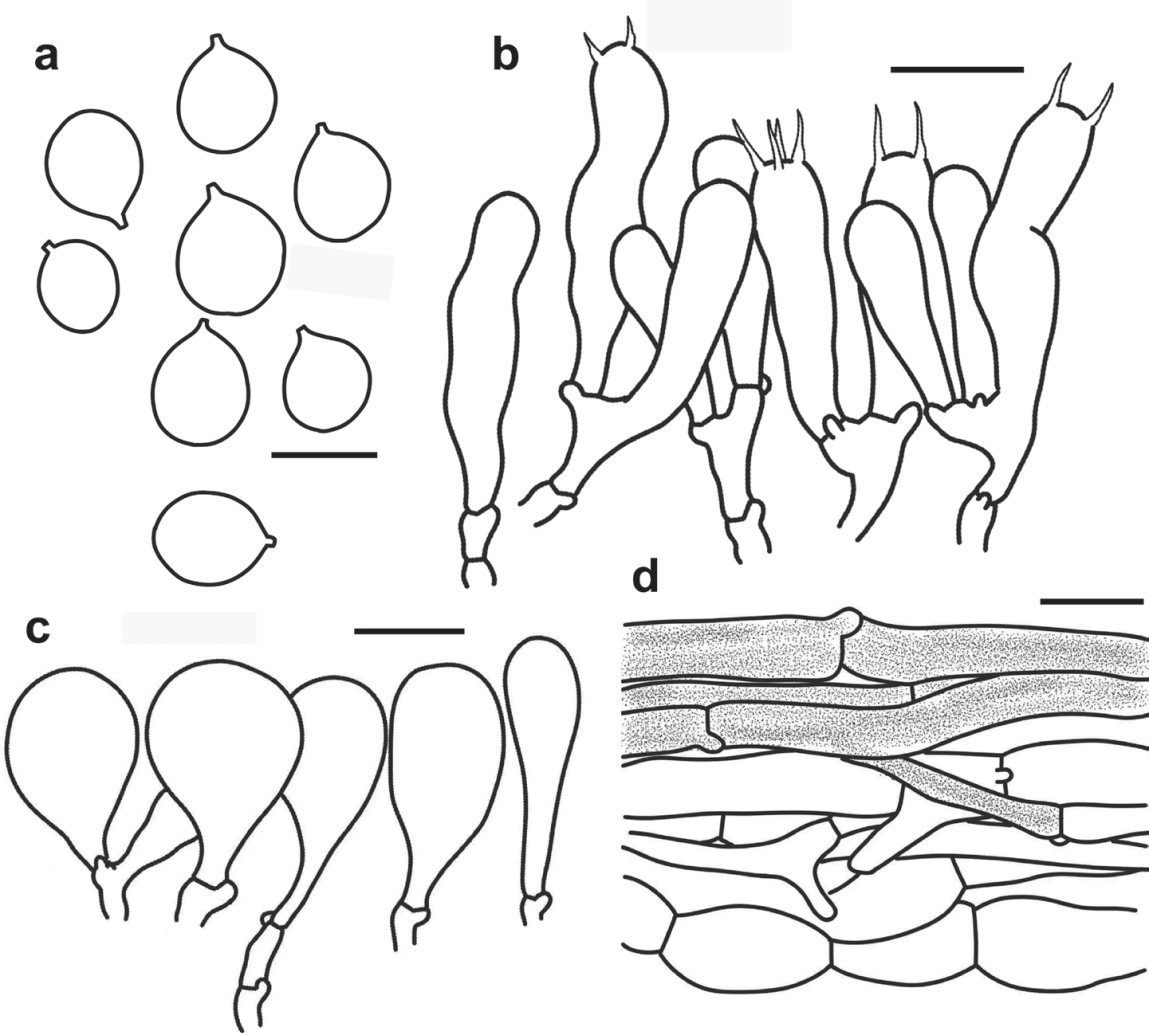
Microscopic features of *Tricholomopsis cortinata* (HKAS 151682, holotype). **a**. Basidiospores; **b**. Hymenium and subhymenium; **c**. Cheilocystidia; **d**. Pileipellis. Scale bars: 5 μm (**a**), 10 μm (**b**), 20 μm (**c, d**).

##### Additional specimen examined.

China, • Yunnan Province: Nujiang Lisu Autonomous Prefecture, Lushui County, Pianma Township, altitude 3141 m, 24 Aug 2020, Yunrui Ma 301 (KUN-HKAS 151681).

##### Notes.

*Tricholomopsis roseorubra* is morphologically most similar to *T. rutilans*, *T. flammula*, *T. pteridiicola*, and *T. depressa* (Smith 1960; Holec and Kolařík 2011; Olariaga et al. 2015; Wang et al. 2024). It resembles *T. rutilans* in having brightly colored basidiomata with a squamulose pileus and stipe but differs in its smaller basidiomata, finer grayish-rose to ruby-red squamules, and cheilocystidia often containing brown vacuolar pigments. It is also similar to *T. flammula* and *T. pteridiicola* in pileus coloration but differs in having a larger pileus with denser and more vividly colored squamules, broader basidiospores, and less frequent pleurocystidia. *T. roseorubra* can be distinguished from *T. depressa* by the absence of a distinctly depressed pileus, ellipsoid to broadly ellipsoid basidiospores, and predominantly clavate cheilocystidia with brown vacuolar pigments, whereas *T. depressa* has more elongate basidiospores, spheropedunculate and typically hyaline cheilocystidia, and more frequent pleurocystidia.

Ecologically, *T. roseorubra* appears to be associated with decaying bamboo leaf litter in montane habitats, which may further distinguish it from most other members of the section.

#### Tricholomopsis cortinata

Taxon classificationFungiAgaricalesTricholomataceae

G.S. Wang, T. Guo & J.P. Liao sp. nov.

A3DF68F6-B4A8-5895-BB69-8D7127A5974A

Fungal Names: FN 573154

##### Etymology.

The epithet “cortinata,” referring to the cortinate annulus on the stipe.

##### Diagnosis.

*Tricholomopsis cortinata* differs from the other species of the genus *Tricholomopsis* by its distinctly smaller basidiomata, small scales on the pileus, cortinate annulus on the stipe, and globose basidiospores.

##### Holotype.

China • Fujian Province: Yongan City, Tianbaoyan National Nature Reserve, altitude 890 m, 18 Jun 2024, Jinpeng Liao 163420 (KUN-HKAS 151682); GenBank ITS: AA136285, 28S: AA136290.

##### Description.

Basidiomata small. Pileus 1–2 cm, plano-convex to applanate, dry, light yellow, yellow to dark yellow (2A6–2A7, 4A5–4A8), ornamented with minute, concolorous scales. Lamellae narrowly adnate to sinuate, up to 1 mm broad, close to crowded, light yellow, yellow to dark yellow (2A6–2A7, 4A5–4A8), with abundant lamellulae. Stipe 1–4 cm long, 0.1–0.3 cm thick, cylindrical, light yellow to yellow (2A6–2A7, 4A5) with minute, pale yellow (1A2–1A3) granules. Partial veil cortinate, white (1A1), membranous-floccose, leaving remnants on the stipe surface as thin, fibrillose-flocculent patches.

Basidiospores [60/3/2] 5.0–5.9(–6.2) × 4.9–5.7(–5.8) μm, Q = (0.91–)1.00–1.06(–1.07), Q_av._ = 1.02±0.03, globose, thin-walled, colorless and hyaline, smooth, non-amyloid; apiculus small. Basidia 15–40 × 5–6 μm, 4-spored or 2-spored, narrowly clavate to flexuose, thin-walled; sterigmata 3–5 μm long. Cheilocystidia 35–40 × 15–32 μm, broadly clavate to spheropedunculate, colorless hyaline, abundant, forming a sterile belt along the lamellar edge. Pleurocystidia absent. Pileipellis a cutis with the transition to a trichoderm at regular intervals, 20–40 μm thick, and composed of radially arranged, thin-walled hyphae 5–12 μm wide and encrusted with brown pigments. Clamp connections present in all parts of basidioma.

##### Habitat and distribution.

Solitary to gregarious on soil among bamboo-*Lauraceae* mixed forests.

##### Additional specimens examined.

China, • Shanghai City, Sheshan National Forest Park, altitude 54 m, 20 Sep 2020, Ting Guo 2115 (KUN-HKAS 151683); • same location, altitude 56 m, 20 Sep 2024, Ting Guo 2120 (KUN-HKAS 151684); • same location, 4 Aug 2021, Ting Guo 2343 (KUN-HKAS 151685).

##### Notes.

*Tricholomopsis cortinata* is morphologically most similar to *T. bambusina* and *T. sasae* within sect. *Bambusinae*. It resembles these species in having yellow-toned basidiomata and a general association with bamboo-related substrates but differs in possessing a well-developed cortinate annulus on the stipe and distinctly smaller basidiomata. Microscopically, *T. cortinata* is distinguished by globose to subglobose basidiospores, whereas those of *T. bambusina* and *T. sasae* are typically ellipsoid. Cheilocystidia in *T. cortinata* are broadly clavate to spheropedunculate and form a well-defined sterile lamellar edge, while those of related species are generally narrower and differ in shape. The presence of a cortinate annulus represents an unusual feature within *Tricholomopsis* and further distinguishes *T. cortinata* from previously described species.

## Discussion

### Phylogenetic placement and ecological differentiation within *Tricholomopsis*

Phylogenetic analyses of the concatenated ITS–28S dataset provide a framework for interpreting morphological and ecological variation within *Tricholomopsis*. The two newly described species are resolved in different sections of the genus, consistent with their contrasting morphologies and ecological preferences. *Tricholomopsis roseorubra* is recovered within sect. *Tricholomopsis*, whereas *T. cortinata* is placed with strong support in sect. *Bambusinae*, which represents the earliest-diverging lineage of the genus in the present phylogeny (Wang et al. 2024).

The placement of *T. roseorubra* within sect. *Tricholomopsis* is consistent with the current circumscription of this section, which comprises species with tricholomoid basidiomata, nonamyloid and smooth basidiospores, prominent cheilocystidia forming a sterile gill edge, and a pileipellis ranging from a cutis to a trichoderm. Ecologically, however, *T. roseorubra* differs from most previously described members of the section by its strict association with dense stands of arrow bamboo at high elevations (ca. 3100 m). In contrast, the majority of species are currently assigned to sect. *Tricholomopsis* are lignicolous or occur on forest litter of coniferous or broad-leaved trees at lower altitudes (Smith 1960; Olariaga et al. 2015; Mao et al. 2021; Liu et al. 2022). This ecological segregation suggests that adaptation to bamboo-dominated subalpine habitats may be associated with ecological differentiation of certain lineages within the section.

By contrast, *T. cortinata* exhibits a combination of characters consistent with sect. *Bambusinae*, including yellow-toned squamulose basidiomata, absence of pleurocystidia, prominent cheilocystidia, and a strong ecological association with bamboo substrates (Wang et al. 2024). These features further support the current delimitation of this section and indicate that species of sect. *Bambusinae* may share a set of morphological and ecological traits linked to bamboo-dominated habitats (Wang et al. 2024).

### Veil evolution and systematic implications within *Tricholomopsis*

The presence of a well-developed cortinate annulus in *Tricholomopsis cortinata* represents a notable departure from the traditional morphological circumscription of *Tricholomopsis*, in which species are generally characterized by the absence of a persistent veil (Smith 1960; Olariaga et al. 2015; Wang et al. 2024). Although annulus-like or fibrillose zones have been reported in some related genera of Phyllotopsidaceae, such as *Conoloma* and *Neotricholomopsis* (Wang and Yang 2023; Fan et al. 2024), these structures are interpreted as fibrillose or annuliform zones rather than true cortinate annuli.

In the present phylogeny, *T. cortinata* is placed within sect. *Bambusinae*, indicating that veil development may occur within this lineage. This observation expands the known morphological variation in *Tricholomopsis* and suggests that veil characters may be more variable than previously recognized. However, given the limited taxon sampling and molecular data currently available, the systematic and evolutionary significance of veil structures in the genus remains unclear and requires further investigation using broader sampling and additional loci.

## Supplementary Material

XML Treatment for Tricholomopsis roseorubra

XML Treatment for Tricholomopsis cortinata

## Additional information

### Artificial Intelligence (AI) use

The authors accept full responsibility for the content of the manuscript, including the disclosure of any use of AI.

No AI tools were used in the preparation of this manuscript.

### Author ORCIDs

G. Wang https://orcid.org/0000-0002-9496-4358

C. Li https://orcid.org/0009-0004-9311-584X

### Data availability

All of the data that support the findings of this study are available in the main text.
